# Eliminating malaria in a sub-Saharan Africa: debate it or just do it?

**DOI:** 10.1186/1475-2875-11-S1-O52

**Published:** 2012-10-15

**Authors:** Richard W Steketee

**Affiliations:** 1 Science Director, Malaria Control Program at PATH

## 

At the outset of the RBM movement in late 1998 with the declared goal of halving the global malaria burden, there was much talk that was eventually (by ~2005) followed by resources and country-led action in scale up of malaria control, notably in sub-Saharan African countries, that has achieved the anticipated burden reduction in many nations. Following the 2007 affirmation that malaria elimination and ultimate eradication was a viable goal, there has been much opportunity for discussion addressing both enthusiasm and caution. That discussion has led to increased clarity on the opportunities to act, and what is meant by “program scale up”, “sustained control”, “pre-elimination” and “elimination”. Yet few sub-Saharan African countries have truly embarked on the needed actions. This talk describes some current country actions, early learning regarding the components of the required actions for sub-Saharan African countries, and discusses details of some critical components including: optimizing preventive intervention coverage; building a sufficient information and surveillance system; using existing tools with updated strategies; measuring and costing; and confronting “time” as both a facilitator and an enemy of progress in elimination.

